# Challenges in the serological evaluation of dogs clinically suspect for canine leishmaniasis

**DOI:** 10.1038/s41598-020-60067-6

**Published:** 2020-02-20

**Authors:** Nuno Santarém, Susana Sousa, Célia G. Amorim, Nuno Lima de Carvalho, Hugo Lima de Carvalho, Óscar Felgueiras, Margarida Brito, Anabela Cordeiro da Silva

**Affiliations:** 1 Instituto de Investigação e Inovação em Saúde, Universidade do Porto, R. Alfredo Allen, 4200-135 Porto, Portugal; 2 Instituto de Biologia Molecular e Celular, Universidade do Porto, R. Alfredo Allen, 4200-135 Porto, Portugal; 3 CEDIVET, Centro de Diagnóstico Veterinário, Rua Antero de Quental, 991, 2°Drt, 4200-071 Porto, Portugal; 4 Departamento de Matemática, Faculdade de Ciências da Universidade do Porto & Centro de Matemática da Universidade do Porto, Rua do Campo Alegre 687, 4150-755 Porto, Portugal; 5 Departamento de Ciências Biológicas, Faculdade de Farmácia da Universidade do Porto, R. Jorge de Viterbo Ferreira 228, 4050-313 Porto, Portugal; 6 Present Address: Faculdade de Ciências da Universidade do Porto, Rua do Campo Alegre 687, 4150-755 Porto, Portugal; 7 Present Address: LAQV-REQUIMTE, Departamento de Química Aplicada, Faculdade de Farmácia da Universidade do Porto, R. Jorge de Viterbo Ferreira 228, 4050-313 Porto, Portugal

**Keywords:** Parasitic infection, Diagnostic markers

## Abstract

Canine leishmaniasis is a major veterinary issue and also a public health challenge due to its zoonotic potential. In this context, serological evaluation is essential for Canine leishmaniasis management. Several serological alternatives, such as rapid diagnostic tests, enzyme-linked immunosorbent assay (ELISA) and immunofluorescence antibody test (IFAT), are well established. In fact, the capacity of distinct tests and antigens, evaluated by their sensitivity and specificity, to detect disease is normally considered sufficient for diagnosing Canine leishmaniasis. In this context, we evaluated the seropositivity using 8 different serological tests (ELISA with *Leishmania* recombinant proteins (rK39, LicTXNPx); soluble promastigote *Leishmania* antigens (SPLA); commercial ELISA test) in 82 clinically suspect animals from Northern Portugal. The obtained serological data originated 50% of inconclusive serological information with a mixture of seropositive and seronegative results for individual animals. Cut-off independent risk groups were then generated from the serological data to evaluate the clustering of the samples. This analysis originated risk groups that correlated with the most seropositive samples, suggesting that this method might be used, in a cut-off independent manner, to improve conventional serological evaluation. Ultimately, given that no test prioritization exists, the use of any single serological test increases the potential for misdiagnosis, along with all associated risks for the dog as well as public health. The use of a cut-off independent analysis has the potential to improve the predictive values of these tests, enabling a more accurate evaluation of the dog’s condition.

## Introduction

Leishmaniasis is a protozoan vector-borne zoonosis caused by several species of *Leishmania* that affects both humans and dogs^1^. Considering the disease’s zoonotic potential, infection control in reservoirs is vital to restrict human zoonotic visceral leishmaniasis. The infection of dogs by *Leishmania infantum* is responsible for a veterinary disease known as Canine leishmaniasis (CanL). The detection of infected animals is a priority to enable appropriate disease containment measures. Quantitative and qualitative serology are considered essential diagnostic tools when used along with clinical signs compatible with CanL^1–3^. Moreover, quantitative serology is important not only for disease diagnosis but also for epidemiological studies enabling the adoption of appropriate CanL containment and control measures^4,5^. Most serological tests present high specificities and sensitivities enabling accurate diagnosis of CanL. Still, comparative studies of serological performance in unbiased clinically suspect animals are lacking. The performance of these tests in field conditions must be addressed, particularly after reports of reduced predictive value of simple serological tests in serological surveys^6^. In this context, we evaluated the consistency of serological evaluation in a group of 82 CanL suspect dogs in Portugal. Eight quantitative serological tests based on immunosorbent assay (ELISA) and immunofluorescence antibody test (IFAT) were used to achieve this objective. In Europe, IFAT is considered the standard method of serological diagnosis of CanL, presenting high sensitivity and specificity (nearly 100% for both) while ELISA is also a quantitative method that allows the use of distinct antigens. In this study, three main antigens were used for the in house ELISA; parasite lysate, rK39 (a reference antigen for serodiagnosis^7^) and LicTXNPX (a protein already evaluated for both human and CanL^8,9^). The tests were performed under the same conditions for all samples to exclude inter-laboratory variability.

## Results

### Seropositivity in clinically suspect dogs

**Figure 1:**
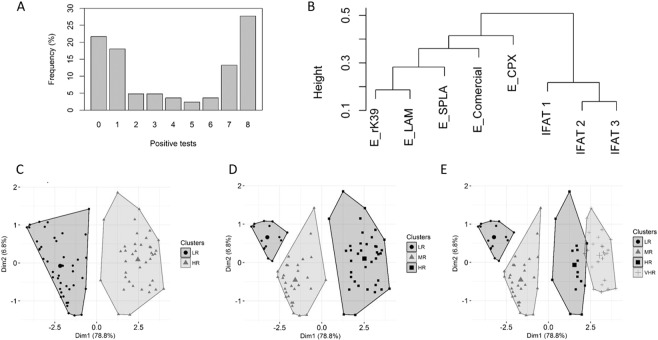
Simple distribution and clusterization of the samples. (**A**) Sample distribution by number of seropositive tests; (**B**) Dendrogram for clustering of markers; (**C**) Clusters and control sample plotted on study cohort based PCA for K2; K3 (**D**) and K4 (**E**).

The serological tests performed produced similar seropositivity levels, showing a strong correlation between all pairs of tests (Table 1). In fact, average seropositivity to the different tests ranged between 42.2 (for E_LicTXNPX) and 57.8% (IFAT). However, only 28% of the samples were positive for all the tests (Fig. 1, Supplemental Table 1), or 39% if we consider only IFAT-based techniques. On the other hand, only 22% were seronegative to all the tests. Therefore, 50% of the cohort presented a combination of seropositive/seronegative results (Fig. 1A). The control samples from CanL animals were all seropositive while the negative samples from a non-endemic area presented only one positive sample for IFAT.

**Table 1:** Overall seropositivity percentage in the cohort and spearman correlation between the different tests performed.

		E_rK39	E_LicTXNPX	E_LAM	E_SPLA	E_Comercial	IFAT 1	IFAT 2	IFAT 3
	Seropositivity (%)	53.0	42.2	56.6	48.2	49.4	57.8	45.8	51.8
Spearman Correlation	E_rK39	1.00	0.83	0.94	0.91	0.79	0.67	0.74	0.80
	E_LicTXNPX	0.83	1.00	0.84	0.84	0.75	0.60	0.64	0.69
	E_LAM	0.94	0.84	1.00	0.87	0.77	0.69	0.75	0.78
	E_SPLA	0.91	0.84	0.87	1.00	0.83	0.72	0.73	0.76
	E_Comercial	0.79	0.75	0.77	0.83	1.00	0.69	0.78	0.78
	IFAT 1	0.67	0.60	0.69	0.72	0.69	1.00	0.69	0.74
	IFAT-2	0.74	0.64	0.75	0.73	0.78	0.69	1.00	0.79
	IFAT-3	0.80	0.69	0.78	0.76	0.78	0.74	0.79	1.00

### Cluster analysis

Considering that no gold standard exists, a cluster model was developed based on the 83 clinically suspect dogs in order to analyse the data (Fig. 1B–E). In the cluster analysis, all serological test values are considered, without considering cut-offs. A split in ELISA and IFAT tests was observed as the two main clusters produced by this algorithm (Fig. 1B). Considering the ELISA branch, E_rk39 and E_LAM are the most similar antigens, whereas E_LicTXNPX2 was the least similar to the others. Conversely, in the IFAT branch, the three markers presented high similarity between one another. This is reflected by the low clustering heights and the high silhouette value of 0.69 (vs. 0.42 for ELISA tests)^10^. The overall silhouette average of 0.52 supports the quality of the obtained clusters.

The data can be presented by means of two components, since it is the smallest number of components that accounts for more than 80% of the variance^11^. Indeed, the first two PCA components, PC1 and PC2, explain 85.5% of the total variance (78.6% for PC1 and 6.8% for PC2). The first component essentially contrasts low and high values for marker tests. The second component contrasts IFAT tests and E_commercial with the remaining ELISA tests. Regarding the number of clusters generated, the elbow method does not indicate a clear choice. However, 2, 3 or 4 clusters seem to be adequate. Therefore, cluster analysis was done using each of these cluster numbers (Fig. 1D,E).

When k = 2, the resulting clusters were labelled as Low Risk (LR) and High Risk (HR). Dogs in HR are those with at least 5 positive test results and a positive CP1 score. Moreover, most of them have 7 or 8 positive tests (34/39). On the other hand, dogs in LR are those with maximum 4 positive tests and a negative CP1 score. The majority of LR has maximum 1 positive test (33/44).

When k = 3, the resulting clusters were labelled as LR, Moderate Risk (MR) and HR. The LR and MR clusters correspond mainly to a split in the LR cluster for k = 2. LR corresponds to lower values for CP1 and higher values for CP2. Furthermore, LR dogs were negative for all markers except for 6 subjects that presented a positive IFAT 1 result.

When k = 4, the resulting clusters were labelled as LR, MR, HR and Very High Risk (VHR). The HR and VHR clusters correspond mainly to a split in the HR cluster for k = 3. The VHR corresponds to higher values for CP1. All the VHR were positive for tests with the exception of three cases: two were negative for CP and one for E_Commercial.

The analysis in the study cohort originated in 46–47% of HR animals, the percentage of seropositive samples for each test in the HR groups when k = 2 and k = 3, depicted in Supplemental File 1. The SPLA was the most represented test in both groups with 95% and 93% of the seropositive samples present in the HR k = 2 and k = 3 groups, respectively. The least represented test was IFAT 1 with only 77% of the seropositive animals included in both HR groups.

The control sample was then used to evaluate the cluster models. In the resulting classification for k = 2, all CanL positive animals are assigned to the HR cluster, while the negative are assigned to LR. For k = 3, all the CanL positive control animals are classified as HR, while 19 of the CanL negative animals are LR and 1 is MR. Finally, when k = 4, all CanL positive animals are in the VHR cluster while 19 of the negative animals are in LR, with one animal in MR.

## Discussion

From the 83 dogs suspected for CanL subjected to the 8 serological evaluations, 28% were seropositive to all tests, 39% if we consider only IFAT based techniques that are often the reference for serological evaluation^4^ (Table 1, Supplemental File 1). Therefore, almost a third of the animals, independent of the test used, were seropositive and confirmed CanL cases. On the other hand, 22% were negative for all tests. In these animals, CanL could be excluded for continuous differential diagnosis. Still, 50% of the animals presented a mixture of seropositive and seronegative data that would require follow-up. In fact, 4% of the animals (3/82) were seropositive for half of the tests. This dispersion in seropositivity was transversal to both ELISA- and IFAT-based approaches. This was unexpected as all the tests present significant correlation between them (Table 1). Significantly this dispersion was absent in the control group used to validate the approach (Supplemental File 1). It should be a cause for concern that tests and antigens that are capable of discriminating diseased animals from healthy animals with more than 90% sensitivity and specificity originated such a high percentage of inconclusive serology^8^. Similar discrepancies between antigens (SPLA and rK39) were reported in the context of non-endemic animals^6^. In fact, complex antigens like SPLA can be associated with cross-reactivity^12,13^. Notwithstanding, the data generated does not suggest lack of specificity as no trend of increased seropositivity was observed when comparing complex antigens like SPLA with recombinant antigens like rK39. In fact, both the spearman correlation and the cluster analysis support the similarity of the seropositive profile between SPLA and rK39. A possible justification for these results is the fact that the cut-off determination is done using animals that are often very sick. This might result in a cut-off that is not adequate for all moments of infection or disease. In fact, it has been proven that serological response to an antigen can show distinct levels during infection, as was demonstrated with experimentally infected animals^14,15^. Therefore, the distinct serological tests might be influenced by this reality as cut-offs are determined using unequivocally diseased animals. These animals have a characteristic serological profile associated with established disease that might not match animals that are at a different stage of disease development^16^. To detect possible similarity patterns between the serological readouts in these animals, a cluster analysis of the tests was performed. These cluster analyses are less influenced by intrinsic cut-offs as they use absolute data and not seropositive/seronegative determination. This analysis grouped the tests into two distinct groups, ELISA- and IFAT-based approaches (Fig. 1B). Considering only the ELISA antigens, rK39 and LAM were most closely clustered. This is expected as rK39 is the main component of LAM^8^. Interestingly, among the ELISA antigens, LicTXNPX presented the lowest correlation with rK39. This is relevant, as the above-mentioned LAM is a mixture of rK39 and LicTXNPX, suggesting that this type of cluster analysis might help in defining the best performing antigens mixtures. In fact, considering the capacity of LicTXNPX to detect subclinical infection in both experimentally infected animals and naturally infected animals with more sensitivity and specificity than rK39 might suggest that these antigens can be recognized in a complementary way^8^. Therefore, this cluster analysis might enable selecting antigens that are complementary and do not overlap in their capacity to provide meaningful serological data. The cluster analysis of the animals made it possible to aggregate dogs into distinct groups (Fig. 1C,D). We may consider that the obtained division into k clusters suggests different levels of diagnosis uncertainty. If k = 2, the clusters seem to provide a reasonable structure since the average silhouette is greater than 0.5 for both (Fig. 1C). For k = 3, the obtained structure with an overall average silhouette of 0.41 appears to be somewhat weaker although still meaningful (Fig. 1D). The LR cluster aggregates dogs with less likelihood of not being infected. The MR cluster forms a group with the lowest average silhouette (0.29). This is in accordance with the fact that it includes 30% of dogs testing negative for all markers and simultaneously 27% of dogs testing positive for at least 3 markers. For k = 4, the overall silhouette average is low (0.29) (Fig. 1E). The cluster corresponding to HR dogs attains a very low silhouette average (0.06) which leads us to conclude that the resulting partition looks artificial. Using the cluster analysis with K = 2 or K = 3, the highest risk groups encompass 46 and 47% of the animals respectively. These percentages are similar to the overall antigen seropositivity obtained for the single antigens (Table 1). Notwithstanding, the representability in the high risk groups was distinct. While 95% of the SPLA seropositive animals were present in the HR groups, only 77% of IFAT 1 seropositive samples were present. This lower seropositivity associated with IFAT 1 was not characteristic of the technical approach as 88–92% of IFAT2 seropositive animals were in the same group.

The two groups, CanL and healthy animals from non-endemic areas, in the control sample were distributed in the opposite risk groups with no overlapping. This clearly demonstrates that the analysis proposed is able to discriminate CanL dogs and that the reported risk groups can contribute to disease staging.

Overall, these clusters generate an interpretable separation that may be useful in accessing not only the individual performance of the different antigens but also in creating serological profiles consistent with potentially distinct clinical settings. Therefore, the obtained data partition highlights the importance of combining results from several markers as opposed to relying on a single one. These approaches might be used to look for specific disease and infection patterns that might enable not only accurate diagnosis but also information about epidemiological relevance concerning subclinical infected reservoirs and the presence of vaccinated animals.

In conclusion, the use of 8 different serological tests in a clinically suspect cohort for CanL, originated 50% of inconclusive serological information with a mixture of seropositive and seronegative data. Considering that no test prioritization exists for half of the animals evaluated, the individual serological data obtained could potentially result in a misdiagnosis with all associated risks to the dog as well as public health, due to the zoonotic potential of the disease. The cut-off independent risk groups correlated with the most seropositive samples enabling the definition of risk groups. In fact, we highlight that diseased and heathy animals from the control sample were placed in the extremities showing their potential for risk staging in CanL. This approach deserves to be explored further by studies with cohorts subjected to longitudinal evaluation.

## Methods

### Canine sera

Dogs living in Northern Portugal that were suspected for CanL after independent veterinarian evaluation (n = 83), displaying signs associated with leishmaniasis: lymphadenomegaly, lymphadenopathy, alopecia, dermatitis, skin ulceration, keratoconjunctivitis, onychogryphosis, lameness, epistaxis, anorexia and weight loss. Group CanL + (n = 20): sera from dogs living in geographical regions of Portugal where CanL is endemic presenting at least two clinical signs compatible with the disease (viz. lymphadenomegaly lymphadenopathy, alopecia, dermatitis, skin ulceration, keratoconjunctivitis, onychogryphosis, lameness, epistaxis, anorexia and weight loss). These animals were also seropositive for anti-*Leishmania* antibodies by the direct agglutination test (DAT) (cut-off titer = 400) and positive for the presence of amastigotes in bone marrow or lymph node aspirates.

Group CanL- (n = 20): sera from dogs that visited a veterinary clinic in a Portuguese region considered to be non-endemic for CanL. All were seronegative by DAT (titer < 100).

This study observed Portuguese legislation for the protection of animals (Law no. 92/1995, from September 12th). According to the European Directive of 24 November 1986, Article 2 d, non-experimental, agricultural and clinical veterinary were excluded.

### Antigens

Four antigens were used for ELISA assays: soluble promastigote *Leishmania* antigen (SPLA), *Leishmania* recombinant proteins - *L*. *infantum* cytosolic tryparedoxin peroxidase (LicTXNPx), and rK39 and LAM, a mixture of LicTXNPx and rK39 previously described by Santarem *et al*.^8^.

All antigens used were quantified by DC^TM^ Protein Assay (BioRad) and stored at −80 °C in single use aliquots.

*Leishmania* promastigotes were obtained as previously described by Santarem *et al*.^8^. Promastigotes were washed three times with phosphate-buffered saline (PBS), pH 7.4 and centrifuged at 3,500 × g, 10 min, 4 °C. Pellet was suspended at a theoretical concentration of 1 × 10^8^ parasites/ml in PBS containing 1 mM phenylmethylsulfonyl fluoride (PMSF) protease inhibitor and submitted to 10 freeze-thaw cycles. The resulting suspension was centrifuged at 13,000 × g for 30 min at 4 °C and the supernatant was recovered (SPLA), quantified and stored.

The gene encoding LicTXNPx protein was cloned in the pET28a vector and the recombinant protein was purified by affinity chromatography on a Ni-NTA column (Qiagen) as described in previous report^17^. The purified molecules were analysed before and after elution on 10% polyacrylamide gels containing 0.2% SDS and visualized by staining with Coomassie blue.

### Enzyme-linked immunosorbent assay (ELISA)

Ninety-six-well flat-bottomed microtiter plates (Greiner Bio-One) were coated with 50 µl of 0.1 M carbonate buffer, pH = 9.6, with SPLA (10 µg/ml), rK39 (5 µg/ml) and LAM (rK39 4 µg/ml + LicTXNPX 1 µg/ml). Plates were incubated ON at 4 °C. The next day, the coating solution was removed and the plates were blocked with 200 µl of PBS-low-fat-milk 3% at 37 °C for 1 h. Next, plates were washed with PBS-Tween 0.05% (PBS-T) and the sera, diluted 1:1500 in PBS-T-low-fat-milk 1%, were dispensed in triplicate (100 µl/well) and incubated at 37 °C for 30 minutes. After another washing step, 100 µl/well of secondary antibody - anti-dog IgG conjugated to horseradish peroxidase (Sigma) - diluted 1:1176.5, was added and the plates were incubated at 37 °C for 30 min. Plates were washed and incubated with 0.5 mg/ml of *о*-phenylenediamine dihydrochloride (Sigma) for 10 min in dark. Reaction was stopped with a 50 µl/well of HCl 3 M. Absorbance was read at 492 nm in an automatic reader (Synergy 2, BioTek Instruments, Inc.).

Leiscan®*Leishmania* ELISA Test (Esteve Veterinaria, Laboratorios Dr. Esteve SA, Spain) was used according the instructions of the supplier.

At least three independent assays performed in triplicate were performed.

### IFAT

1. Fluoleish (Virbac BVT) was used for the detection of anti-*Leishmania infantum* antibodies using a fluorescein-labelled antiglobulin (antigen of *Leishmania infantum* zymodeme Mon 1) according to the instructions of the supplier.
2. Homemade IFAT Individual slide wells were covered with 20 µl of 4 × 10^8^ *Leishmania* promastigotes suspension. After drying, slides were fixed with cold acetone and left drying again for 30 minutes. Four serial dilutions (1/20, 1/40, 1/80 and 1/160) were made for each serum and 15 µl of each dilution were dispensed in the slides. After 30 minutes in a humid chamber at 37 °C, slides were washed with PBS and H_2_0 and left drying at room temperature (RT). Anti-dog IgG with Evan´s blue was added to slides (15 µl/well) and left in humid chamber at 37 °C. Slides were washed with PBS and H_2_0 and left drying at RT. Cover slips were mounted and slides observed by fluorescence microscopy.
3. MegaFLUO LEISH (MEGACOR Diagnostik, GmbH) was outsourced and performed according to the instructions of the supplier.

At least three independent assays were performed.

### Data analysis and statistical methods

The seropositivity cut-off values used for the ELISA antigens, rk39, SPLA, LicTXNPx and LAM were reported previously by Santarem *et al*.^8^, positivity to commercial tests was determined according to the manufacturer instructions. The average of at least three independent assays was used for all the subsequent analysis. Log transformation was applied to ELISA test results to reduce right skewness. In order to compare the different diagnostic tests, all test data were then scaled. The correlation between the tests was analysed through Spearman’s rank correlation coefficient. In this work, a gold standard is not assumed and the similarities between the tests are further explored by performing a hierarchical cluster analysis, using the Agnes algorithm. This is an agglomerative method starting with all 8 markers as clusters that proceeds by successive fusions until a single cluster is obtained. A dendrogram of the cluster linkage between markers was constructed based on a dissimilarity matrix with Gower coefficients and the average method, where the distance between two clusters is the average of the dissimilarities between the elements of one cluster and those from another cluster. For establishing a diagnosis differentiation of the dogs, a cluster analysis of the dogs was performed, using all the markers results. Similar dogs were grouped using the k-means method. In this analysis the number of clusters, k, must be fixed in advance and each observation is assigned to exactly one cluster. Random centers are initially chosen for each cluster. Then, each observation is assigned to the cluster whose center is closest with respect to the Euclidean distance. Next, cluster centers are updated by taking the position of the mean of their members (centroids). This procedure is repeated until centroids become stable. The resulting clusters depend on the initially chosen centers. As such the algorithm was run 25 times and selected the partition minimizing the total sum of squared distances to centroids. The silhouette coefficient values are presented for showing the clustering structures’ strength. Principal component analysis (PCA) was then applied in order to graphically represent the data and their clusters. The so fitted models were then evaluated by classifying the dogs of the control sample, through their assignment to the cluster with the nearest centroid. Statistical analysis was performed using the cluster and factoextra packages implemented in R^18^.

## Supplementary information

Supplementarytable 1.
