# Author Correction: Challenges in the serological evaluation of dogs clinically suspect for canine leishmaniasis

**DOI:** 10.1038/s41598-020-66088-5

**Published:** 2020-05-28

**Authors:** Nuno Santarém, Susana Sousa, Célia G. Amorim, Nuno Lima de Carvalho, Hugo Lima de Carvalho, Óscar Felgueiras, Margarida Brito, Anabela Cordeiro da Silva

**Affiliations:** 1 Instituto de Investigação e Inovação em Saúde, Universidade do Porto, R. Alfredo Allen, 4200-135 Porto, Portugal; 2 Instituto de Biologia Molecular e Celular, Universidade do Porto, R. Alfredo Allen, 4200-135 Porto, Portugal; 3 CEDIVET, Centro de Diagnóstico Veterinário, Rua Antero de Quental, 991, 2°Drt, 4200-071 Porto, Portugal; 4 Departamento de Matemática, Faculdade de Ciências da Universidade do Porto & Centro de Matemática da Universidade do Porto, Rua do Campo Alegre 687, 4150-755 Porto, Portugal; 5 Departamento de Ciências Biológicas, Faculdade de Farmácia da Universidade do Porto, R. Jorge de Viterbo Ferreira 228, 4050-313 Porto, Portugal; 6 Present Address: Faculdade de Ciências da Universidade do Porto, Rua do Campo Alegre 687, 4150-755 Porto, Portugal; 7 Present Address: LAQV-REQUIMTE, Departamento de Química Aplicada, Faculdade de Farmácia da Universidade do Porto, R. Jorge de Viterbo Ferreira 228, 4050-313 Porto, Portugal

Correction to: *Scientific Reports* 10.1038/s41598-020-60067-6, published online 20 February 2020

In Figure 1C, D, and E the positive and negative controls are missing. The correct Figure 1 appears below.

**Figure 1:**
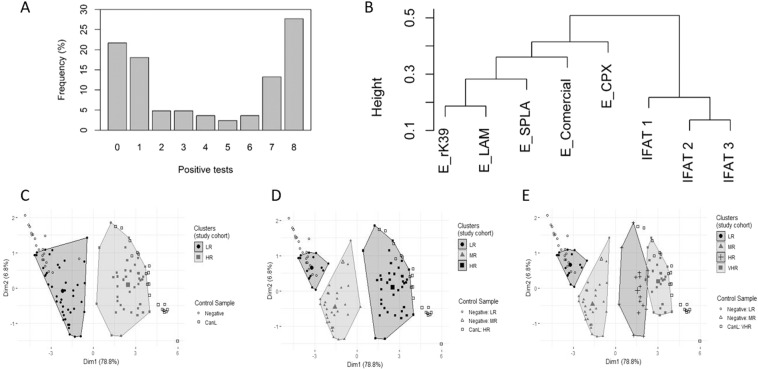
.

