# Supplementary material for: Challenges in the serological evaluation of dogs clinically suspect for canine leishmaniasis
Source: Sci Rep. 2020 Feb 20;10:3099. doi: 10.1038/s41598-020-60067-6 (PMC7033258; doi:10.1038/s41598-020-60067-6)
Supplement: Supplementary file 1 — Supplementarytable 1. [file 41598_2020_60067_MOESM1_ESM.pdf]

Challenges in the serological evaluation of dogs clinically suspect for canine leishmaniasis

Nuno Santarém[1,2], Susana Sousa[1,2,§,] Célia G. Amorim[1,2#], Nuno Lima de Carvalho[3,] Hugo Lima de Carvalho[3] ,Óscar Felgueiras[4], Margarida Brito[4*], Anabela Cordeiro da Silva[1,2,5*]

1Instituto de Investigação e Inovação em Saúde, Universidade do Porto, R. Alfredo Allen, 4200-135 Porto, Portugal
2Instituto de Biologia Molecular e Celular, Universidade do Porto, R. Alfredo Allen, 4200-135 Porto, Portugal
3CEDIVET, Centro de Diagnóstico Veterinário, Rua Antero de Quental, 991, 2ºDrt, 4200-071 Porto, Portugal
4 Departamento de Matemática, Faculdade de Ciências da Universidade do Porto & Centro de Matemática da Universidade do Porto, Rua do Campo Alegre 687, 4150-755 Porto, Portugal
5Departamento de Ciências Biológicas, Faculdade de Farmácia da Universidade do Porto, R. Jorge de Viterbo Ferreira 228, 4050-313 Porto, Portugal

§ Present address: Faculdade de Ciências da Universidade do Porto, Rua do Campo Alegre 687, 4150-755 Porto, Portugal.
# Present address: LAQV-REQUIMTE, Departamento de Química Aplicada, Faculdade de Farmácia da Universidade do Porto, R. Jorge de Viterbo Ferreira 228, 4050-313, Portugal.

ACS: phone number: +351966125016, cordeiro@ff.up.pt or cordeiro@ibmc.up.pt (*Corresponding author)
MB: phone number: +351 220402253, mabrito@fc.up.pt (*Co-corresponding author)

Supplemental table 1: Average raw data and respective attributed cluster. Raw data represents the average from as least three independent experiments. Cut offs are depicted above the column for each antigen. Cells in green represent seropositive tests while red represent seronegative. The atributed cluster each sample in the K2, K3 and K4 analysis is also depicted.

		ELISA					IFAT			Attributed Cluster		
	Cut-off	0,094	0,146	0,149	0,156	1,1	<1/40	<1/40	<1/40			
	Test name	rK39	CPX	LAM	SPLA	Esteve	Fluoleish	In house	External			
Sample	ID	E_rk39	E_, LicTXNPx	E_LAM	E_SPLA	E_Commercial	IFAT_1	IFAT_2	IFAT_2	K2	K3	K4
1	11/09411	0,022	0,068	0,159	0,051	0,271	<1/20	<1/20	<1/32	1	2	2
2	11/09616	0,017	0,037	0,061	0,107	0,397	<1/20	<1/20	<1/32	1	2	2
3	11/09418	1,225	0,123	1,913	0,418	1,711	³1/160	1/80	1/64	2	3	3
4	11/09626	0,013	0,032	0,068	0,048	0,194	³1/160	<1/20	<1/32	1	1	1
5	11/09649	1,412	0,233	2,380	0,427	4,405	³1/160	³1/160	1/1024	2	3	4
7	11/09724	1,410	0,210	1,838	0,761	4,321	³1/160	>1/160	1/1024	2	3	4
8	11/10014	1,187	1,111	1,456	0,765	5,482	³1/160	³1/160	1/512	2	3	4
9	11/10022	0,027	0,224	0,084	0,144	3,451	1/80	³1/160	1/64	2	3	3
10	11/10321	0,017	0,022	0,055	-0,004	0,151	1/80	<1/20	<1/32	1	1	1
11	11/10671	0,024	0,050	0,130	0,002	0,237	1/40	<1/20	1/32	1	1	1
12	11/10724	0,039	0,065	0,153	0,011	0,282	<1/20	<1/20	1/32	1	2	2
13	11/10863	1,655	0,718	2,402	0,689	5,051	³1/160	>1/160	1/2048	2	3	4
14	11/11106	1,314	0,912	1,638	0,882	4,847	³1/160	³1/160	1/32	2	3	4
15	11/11309	0,037	0,070	0,163	0,049	0,238	<1/20	<1/20	<1/32	1	2	2
16	11/11310	0,023	0,060	0,123	0,026	0,184	<1/20	1/80	<1/32	1	2	2
17	11/11679	0,084	0,058	0,106	0,089	0,247	1/80	<1/20	<1/32	1	2	2
18	11/11699	0,095	0,167	0,108	0,120	0,421	1/40	<1/20	1/64	1	2	2
19	11/11764	0,382	0,084	0,637	0,168	0,923	<1/20	1/40	1/256	2	2	3
20	11/11820	0,046	0,042	0,082	0,068	2,461	<1/20	1/80	1/1024	1	2	2
21	11/12017	2,297	0,297	2,094	1,434	5,182	<1/20	³1/160	<1/32	2	3	3
22	11/12032	0,085	0,062	0,182	0,242	2,127	³1/160	³1/160	1/1024	2	3	3
23	11/12071	0,013	0,031	0,062	0,033	0,145	1/80	<1/20	<1/32	1	1	1
24	11/12252	0,018	0,025	0,065	0,013	0,272	<1/20	<1/20	<1/32	1	1	1
26	11/12703	0,021	0,059	0,082	0,022	1,112	<1/20	<1/20	1/32	1	2	2
27	11/12704	0,242	0,675	0,555	0,223	1,510	³1/160	³1/160	1/128	2	3	3

28	11/12815	0,048	0,068	0,251	0,088	0,174	1/80	<1/20	<1/32	1	2	2
29	11/12843	0,009	0,015	0,023	0,016	0,320	<1/20	<1/20	<1/32	1	1	1
32	11/13809	0,128	0,059	0,191	0,127	0,178	<1/20	<1/20	<1/32	1	2	2
34	11/14529	0,046	0,031	0,050	0,102	1,990	^{3}1/160	1/20	1/128	1	2	2
35	11/14643	0,806	0,114	0,871	0,265	3,076	^{3}1/160	1/160	1/1024	2	3	4
36	11/15139	2,086	1,233	2,202	0,495	3,714	^{3}1/160	>1/160	1/1024	2	3	4
37	11/15423	0,458	0,434	0,519	0,359	3,483	^{3}1/160	1/80	<1/32	2	3	4
38	11/16328	1,407	0,755	1,758	0,559	4,112	^{3}1/160	>1/160	1/512	2	3	4
39	11/16402	0,142	0,040	0,180	0,139	0,276	<1/20	<1/20	1/32	1	2	2
40	11/16437	0,931	0,074	1,223	0,309	3,616	^{3}1/160	>1/160	1/1024	2	3	4
41	11/16449	0,268	0,118	0,349	0,222	0,195	<1/20	<1/20	1/64	1	2	2
42	11/16717	0,515	0,126	0,786	0,834	2,046	^{3}1/160	1/80	<1/32	2	3	3
43	11/16727	0,044	0,035	0,069	0,040	0,484	<1/20	<1/20	<1/32	1	2	2
44	11/17702	0,483	0,096	0,668	0,473	2,123	1/80	1/40	1/512	2	3	3
45	11/20197	0,001	0,034	0,017	0,001	0,264	<1/20	<1/20	< 1/32	1	1	1
46	11/20198	1,201	0,765	1,030	1,253	4,206	^{3}1/160	^{3}1/160	1/256	2	3	4
47	11/21307	0,108	0,137	0,133	0,310	0,322	<1/20	<1/20	< 1/32	1	2	2
48	12/01056	2,597	3,707	2,438	3,666	2,600	^{3}1/160	^{3}1/160	1/1024	2	3	4
49	12/01070	0,398	2,169	0,443	0,322	0,431	^{3}1/160	^{3}1/160	1/1024	2	3	4
50	12/01153	0,725	2,873	2,148	0,508	2,131	1/80	1/80	1/512	2	3	4
51	12/01403	0,043	0,069	0,071	0,086	0,826	<1/20	<1/20	< 1/32	1	2	2
52	12/01432	0,038	0,045	0,060	0,024	0,206	<1/20	<1/20	< 1/32	1	2	2
54	12/03540	0,077	0,178	0,088	0,066	0,374	<1/20	<1/20	< 1/32	1	2	2
55	12/03657	1,145	0,229	1,097	0,438	3,967	≥1/160	<1/20	1/256	2	3	3
56	12/03824	1,059	0,865	1,137	1,185	4,739	≥1/160	≥1/160	1/1024	2	3	4
57	12/03855	0,262	0,719	0,598	0,720	3,755	≥1/160	1/80	1/512	2	3	4
58	12/03981	0,004	0,009	0,004	0,013	0,319	<1/20	<1/20	< 1/32	1	1	1
59	12/04024	0,041	0,176	0,078	0,132	1,539	1/80	1/20	< 1/32	1	2	2
60	12/04025	0,101	0,163	0,184	0,121	1,017	<1/20	1/20	1/64	1	2	2
61	12/04027	0,008	0,017	0,007	0,014	0,168	1/40	<1/20	< 1/32	1	1	1
62	12/04183	0,010	0,011	0,019	0,026	0,204	<1/20	<1/20	< 1/32	1	1	1
63	12/04184	0,056	0,493	0,127	0,043	0,250	<1/20	<1/20	< 1/32	1	2	2
64	12/04196	1,107	1,128	1,313	1,065	7,816	≥1/160	≥1/160	1/1024	2	3	4

65	12/04480	0,054	0,063	0,035	0,067	0,450	<1/20	<1/20	1/32	1	2	2
66	12/04531	0,188	0,050	0,239	0,028	0,300	<1/20	1/160	< 1/32	1	2	2
67	12/05823	0,010	0,012	0,010	0,033	0,222	1/40	<1/20	< 1/32	1	1	1
68	12/05824	0,009	0,007	0,006	0,008	0,210	<1/20	<1/20	< 1/32	1	1	1
69	12/05956	1,121	0,111	1,128	0,872	3,942	≥1/160	1/160	1/128	2	3	3
70	12/06141	0,016	0,014	0,010	0,025	0,195	<1/20	<1/20	< 1/32	1	1	1
71	12/06142	0,068	0,074	0,067	0,095	0,163	<1/20	<1/20	< 1/32	1	2	2
72	12/06143	1,251	0,590	1,123	0,859	5,282	≥1/160	1/160	1/1024	2	3	4
73	12/06455	1,352	0,220	1,241	0,597	3,805	≥1/160	1/80	1/1024	2	3	4
74	12/07010	0,059	0,123	0,074	0,083	0,286	<1/20	<1/20	1/64	1	2	2
75	12/08447	0,019	0,008	0,016	0,012	0,203	<1/20	<1/20	1/32	1	1	1
76	12/08573	1,562	0,304	1,579	0,918	2,293	1/40	1/160	1/1024	2	3	4
77	12/08796	0,737	0,875	1,577	0,562	3,077	≥1/160	1/20	1/256	2	3	3
79	12/08838	0,057	0,130	0,116	0,089	0,277	<1/20	<1/20	< 1/32	1	2	2
80	12/08870	0,013	0,026	0,021	0,023	0,201	<1/20	<1/20	< 1/32	1	1	1
81	12/09049	1,414	0,283	1,568	0,794	2,334	≥1/160	1/40	1/64	2	3	3
82	12/09458	0,070	0,094	0,064	0,084	0,265	<1/20	<1/20	< 1/32	1	2	2
83	12/09625	1,639	1,872	2,153	1,121	4,928	≥1/160	1/80	1/512	2	3	4
84	12/09737	2,432	1,370	2,480	1,428	3,340	≥1/160	1/40	1/512	2	3	4
85	12/09941	0,475	1,416	1,099	0,306	1,722	1/40	<1/20	1/256	2	3	3
86	12/10016	0,016	0,033	0,041	0,063	0,303	<1/20	<1/20	< 1/32	1	2	2
87	12/10067	0,123	1,548	0,930	0,689	4,958	≥1/160	1/40	1/32	2	3	3
88	12/10469	1,882	2,246	2,190	1,827	6,239	≥1/160	1/40	1/1024	2	3	4
89	12/10492	0,856	0,120	1,653	0,834	3,279	≥1/160	1/20	1/128	2	3	3
90	12/10538	1,346	1,542	2,197	1,720	6,438	≥1/160	1/160	1/64	2	3	4
101	CanL (+)	0,477	0,161	0,137	0,180	3,485	>1:160	>1:160	1:1024	2	3	4
102	CanL (+)	0,397	1,709	1,307	3,136	7,737	>1:160	>1:160	1:1024	2	3	4
103	CanL (+)	0,669	1,531	0,117	3,146	5,242	>1:160	>1:160	1:1024	2	3	4
104	CanL (+)	0,804	3,213	1,606	2,357	4,273	>1:160	>1:160	1:1024	2	3	4
105	CanL (+)	0,383	1,280	0,053	1,760	2,939	>1:160	>1:160	1:1024	2	3	4
106	CanL (+)	0,993	3,528	0,067	1,804	3,960	>1:160	>1:160	1:1024	2	3	4
107	CanL (+)	1,689	23,215	0,063	3,891	8,081	>1:160	>1:160	1:1024	2	3	4

108	CanL (+)	1,462	3,570	0,386	2,912	8,475	>1:160	>1:160	1:1024	2	3	4
109	CanL (+)	2,615	24,195	3,382	3,618	7,242	>1:160	>1:160	1:1024	2	3	4
110	CanL (+)	0,180	0,198	0,148	0,152	1,404	>1:160	>1:160	1:1024	2	3	4
111	CanL (+)	3,198	36,905	2,164	3,127	13,566	>1:160	>1:160	1:1024	2	3	4
112	CanL (+)	1,466	3,302	2,124	3,293	2,667	>1:160	>1:160	1:1024	2	3	4
113	CanL (+)	3,294	9,345	3,703	11,715	12,242	>1:160	>1:160	1:1024	2	3	4
114	CanL (+)	1,934	15,939	2,326	17,194	7,505	>1:160	>1:160	1:1024	2	3	4
115	CanL (+)	2,149	16,432	2,347	21,169	14,394	>1:160	>1:160	1:1024	2	3	4
116	CanL (+)	1,309	2,596	1,278	2,669	5,273	>1:160	>1:160	1:1024	2	3	4
117	CanL (+)	2,958	13,136	2,620	13,613	7,444	>1:160	>1:160	1:1024	2	3	4
118	CanL (+)	2,114	12,509	1,252	12,900	3,909	>1:160	>1:160	1:1024	2	3	4
119	CanL (+)	0,584	3,072	0,091	1,623	1,333	>1:160	>1:160	1:1024	2	3	4
120	CanL (+)	1,813	14,388	42,896	40,151	14,949	>1:160	>1:160	1:1024	2	3	4
121	Healthy	0,008	0,008	0,025	0,026	0,606	<1:20	<1:20	< 1:32	1	1	1
122	Healthy	0,004	0,010	0,011	0,022	0,485	<1:20	<1:20	< 1:32	1	1	1
123	Healthy	0,006	0,013	0,007	0,012	0,485	<1:20	<1:20	< 1:32	1	1	1
124	Healthy	0,011	0,013	0,008	0,018	0,586	<1:20	<1:20	< 1:32	1	1	1
125	Healthy	0,011	0,008	0,022	0,008	0,515	<1:20	<1:20	< 1:32	1	1	1
126	Healthy	0,015	0,012	0,028	0,018	0,556	<1:20	<1:20	1:32	1	1	1
127	Healthy	0,002	0,005	0,002	0,005	0,455	<1:20	<1:20	< 1:32	1	1	1
128	Healthy	0,002	0,014	0,016	0,017	0,465	<1:20	<1:20	< 1:32	1	1	1
129	Healthy	0,001	0,008	0,014	0,031	0,465	1:40	<1:20	< 1:32	1	1	1
130	Healthy	0,002	0,003	0,005	0,014	0,545	<1:20	<1:20	< 1:32	1	1	1
131	Healthy	0,005	0,004	0,001	0,001	0,475	<1:20	<1:20	< 1:32	1	1	1
132	Healthy	0,006	0,002	0,004	0,003	0,495	<1:20	<1:20	< 1:32	1	1	1
133	Healthy	0,002	0,007	0,013	0,009	0,576	<1:20	<1:20	< 1:32	1	1	1
134	Healthy	0,016	0,023	0,118	0,112	1,010	1:40	1:40	1:64	1	2	2
135	Healthy	0,003	0,001	0,010	0,004	0,778	<1:20	<1:20	< 1:32	1	1	1
136	Healthy	0,001	0,003	0,031	0,021	0,778	<1:20	<1:20	1:32	1	1	1
137	Healthy	0,001	0,001	0,011	0,011	0,606	<1:20	<1:20	< 1:32	1	1	1
138	Healthy	0,003	0,005	0,012	0,004	0,525	<1:20	<1:20	< 1:32	1	1	1

139	Healthy	0,001	0,010	0,012	0,046	0,828	<1:20	<1:20	< 1:32	1	1	1
140	Healthy	0,001	0,001	0,006	0,006	0,667	<1:20	<1:20	< 1:32	1	1	1
